# Relationship between lactate and thiamine-responsive disorders in hospitalised infants and children in Lao PDR: secondary analysis of a prospective cohort study

**DOI:** 10.1080/20469047.2024.2421624

**Published:** 2024-11-07

**Authors:** Kristin Cardiel Nunez, Sonja Y. Hess, Charles D. Arnold, Taryn J. Smith, Indi Trehan, Laurent Hiffler, Dalaphone Sitthideth, Kerry S Jones, Sengchanh Kounnavong, Philip R. Fischer

**Affiliations:** a Pediatrics Residency Program, Children’s Hospital of Philadelphia, Philadelphia, Pennsylvania, USA;; b Institute for Global Nutrition and Department of Nutrition, University of California Davis, Davis, California, USA;; c Departments of Pediatrics, Global Health, and Epidemiology, University of Washington, Seattle, Washington, USA;; d Independent Pediatrician, Lagny sur Marne, France;; e Lao Tropical and Public Health Institute, Vientiane, Lao People’s Democratic Republic;; f Nutritional Biomarker Laboratory, MRC Epidemiology Unit, University of Cambridge, Cambridge, UK;; g Pediatric and Adolescent Medicine, Mayo Clinic, Rochester, Minnesota, USA; Khalifa University College of Medicine and Health Sciences, Abu Dhabi, United Arab Emirates

**Keywords:** Thiamine, thiamine deficiency, beriberi, lactate, hyperlactataemia

## Abstract

**Background:**

Lactate is a by-product of thiamine-deficient cellular metabolism, and hyperlactataemia can indicate severe illness. However, little is known about the clinical significance of hyperlactataemia in thiamine deficiency disorders (TDDs).

**Aim:**

To describe the relationship between whole blood lactate level and thiamine-responsive disorders (TRDs) in children with signs/symptoms of thiamine deficiency in a high-risk region.

**Methods:**

This is a secondary analysis of data from the Lao Thiamine study, a prospective cohort study which enrolled hospitalised infants and children (aged 21 days to <18 months of age) who had at least one sign or symptom suggestive of thiamine deficiency in Lao PDR. Therapeutic thiamine was administered, and clinical evaluations were completed at several time-points over the next 72 hours. Three paediatricians reviewed individual case reports to evaluate clinical response to thiamine and assigned TRD status. Here, data from 402 children were analysed with logistic regression and predictive modelling to examine the relationship between hyperlactataemia and TRDs.

**Results:**

Baseline hyperlactataemia (lactate >4.0 mmol/L) was associated with increased odds of clinical improvement after thiamine administration [OR (95% CI) 2.32 (1.28–4.45), *p*=0.007]. Baseline hyperlactataemia was a significant predictor of thiamine deficiency [thiamine diphosphate (ThDP) <40 nmol/L] [area under the receiver operating curve (95% CI) 0.76 (0.67–0.84), *p*<0.001], and increased odds of mortality [OR (95% CI) 3.51 (1.38–8.94), *p*=0.009].

**Conclusions:**

In children with signs/symptoms of thiamine deficiency, hyperlactataemia is associated with a favourable clinical response to thiamine, biochemical thiamine deficiency, and increased odds of mortality. Lactate may be useful in identifying children who might benefit from therapeutic thiamine administration.

## Introduction

Blood lactate level is used clinically to inform differential diagnoses, predict outcome in severe illness, and monitor response to treatment [1]. While some studies conclude that hyperlactataemia is a predictor of poor outcome from undifferentiated illnesses, others suggest that the usefulness of lactate as a prognostic indicator is dependent on the illness aetiology [2–6].

One aetiology of hyperlactataemia is thiamine deficiency. Thiamine diphosphate (ThDP), the active form of thiamine (vitamin B_1_), is an essential co-factor for enzymes of aerobic metabolism, including pyruvate dehydrogenase complex and alpha-ketoglutarate [7]. Thiamine deficiency results in suboptimal function of enzymes, leading to accumulation of pyruvate and conversion to lactate in anaerobic metabolism [7].

The clinical manifestations of thiamine deficiency, also termed thiamine deficiency disorders (TDDs), of which beriberi is the most severe form, are classically categorised into two subtypes based on symptomatology. First, ‘wet’ beriberi describes predominantly cardiac manifestations, including right heart failure, pulmonary oedema and hepatic congestion, and is thought to present primarily in infants. ‘Dry’ beriberi describes neurological manifestations such as muscle weakness and neuropathy, and is thought to occur primarily in adults [8]. However, a third presentation, Shoshin beriberi, is hallmarked by fulminant cardiovascular collapse with cardiogenic shock and hyperlactataemia [9]. Despite use of hyperlactataemia as a predictor of morbidity and mortality in a variety of critical illnesses, and as a marker of a specific beriberi subtype, little is known about hyperlactataemia in the broader spectrum of TDDs.

The present paper is a secondary analysis of the Lao Thiamine Study, the primary purpose of which was to develop a predictive model for thiamine responsive disorders (TRDs) in infants and young children with signs and symptoms suggestive of clinical thiamine deficiency [10]. In the Lao Thiamine Study, of the 449 children with a median (IQR) age of 2.9 (4.0) months, 60.8% had a TRD [11]. The relationship between baseline blood lactate levels and TRDs is examined here.

## Methods

### Data source

This is a secondary analysis of data collected as part of the Lao Thiamine study, a prospective cohort study which enrolled infants and young children hospitalised for the management of symptoms consistent with thiamine deficiency at Lao Friends Hospital for Children, Luang Prabang, northern Lao People’s Democratic Republic (Lao PDR). That study’s protocol, methods and results have been reported elsewhere [10,11]. Briefly, children eligible for study enrollment were those aged 21 days to <18 months with at least one sign or symptom suggesting thiamine deficiency for whom there was written parental consent (Supplemental Table 1). The study was conducted from June 2019 to the end of December 2020.

The Lao Thiamine Study was approved by the National Ethics Committee for Health Research, Ministry of Health, Lao PDR, and by the Institutional Review Board (IRB) of the University of California, Davis. The study was registered at clinicaltrials.gov (NCT03626337). Because of the design of this study as a secondary analysis of de-identified, existing data collected through the approved Lao Thiamine Study, it was deemed to be exempt from IRB review by the Mayo Clinic IRB (45 CFR 46.104.D.4.ii).

### Assessment of clinical response to thiamine

In the Lao Thiamine Study, enrolled children received 100 mg parenteral thiamine per day for at least 3 days, alongside any other treatment prescribed by the hospital physicians. Physical examination and assessment of recovery were done frequently for 72 hours after the initiation of thiamine supplementation [10]. Individual case reports were generated for each child with at least 12 hours of observation. An example case report is available online [12]. A child’s clinical response to therapeutic thiamine administration, the basis of TRD status assignment, was determined by a panel of three expert paediatricians who specialised in tropical medicine. Based on careful review of the timing and extent of changes in general status and specific clinical factors, the paediatricians subjectively assessed whether or not the child had responded to the administration of thiamine. Factors in case assignment included a child’s age, presenting symptoms, evolution of vital signs and symptoms, clinical course within 4–12 hours of receiving therapeutic thiamine, consideration of any other aetiology for the clinical presentation, and cardiac and cranial ultrasound imaging reports. They did not have access to the lactate concentrations during review of the case reports. One of four possible statuses was assigned to each child: classical beriberi, probable TRD, possible TRD and not likely TRD. In cases of initial discordant case assignment, final assignment was determined the panel of expert paediatricians until a consensus was reached as far as possible. The overall agreement rate was greater than 95% [11]. In four cases, however, a consensus could not be reached, and so they are not included in the TRD analyses. Finally, a child was classified as ill with a TRD if assigned to a status of either classical beriberi or probable TRD. A child was classified as ill with a non-TRD if assigned to a status of either possible TRD or not likely TRD.

### Biomarker data

Blood sample collection and laboratory analyses have been detailed previously [10]. Briefly, lactate concentration (StatStrip Lactate, Nova Biomedical, Waltham, MA, USA) was determined immediately after venous blood was collected. While hyperlactataemia can be defined as a lactate level of >2.0 mmol/L, severe-range lactate with widely accepted clinical significance and evidence of metabolical disturbance with or without detectable acidosis is defined as >4.0 mmol/L [13,14]. Hyperlactataemia is defined here as severe-range lactate (>4.0 mmol/L) and reference range lactate as ≤4.0 mmol/L.

All blood samples were stored at −80°C. Samples were batch-shipped from Lao Friends Hospital for Children on dry ice to the University of California Davis, and then to collaborating laboratories for analysis. Biomarkers of thiamine status, including thiamine diphosphate (ThDP) and erythrocyte transketolase activity coefficient (ETKac), were analysed at the Nutritional Biomarker Laboratory, University of Cambridge, UK. ETKac was calculated from an assay which monitors the rate of oxidation of NADH according to previously published methods [15]. ETKac >1.25 U/g Hb was considered to indicate thiamine deficiency [15]. Whole-blood ThDP was measured using the thiochrome reaction coupled with high-performance liquid-chromatography fluorescence detection [16]. While there is no universal consensus regarding a ThDP level which defines thiamine deficiency, some studies evaluating healthy individuals use a cut-off of ThDP <70 nmol/L [16,17]. A greater degree of ThDP deficiency has been associated with more severe clinical phenotypes such as cardiac changes on echocardiography, and so a more conservative cut-off of <40 nmol/L was used in this study [18].

### Statistical analyses

A statistical analysis plan was developed and published prior to analysis [19]. Here, baseline characteristics, including age, sex and ethnicity are presented as medians and proportions for continuous and categorical variables, respectively. Distribution of biochemical variables was skewed, and so they are presented as median and interquartile range (IQR). Means are reported for clinical usefulness. Differences in two reported medians were evaluated by the Mann–Whitney U-test and means by Student’s *t*-test. The primary outcome was the binary variable TRD status.

Logistic regression was used to examine the relationship between predictor variables, including continuous lactate and binary lactate, and the binary outcome of TRD status. Univariate models were adjusted by age, sex, region, ethnic group and carer-reported recent vomiting, diarrhoea, illness or use of medication for an acute illness in the mother or child (separately) in the 2 weeks before hospitalisation. Significant predictors were evaluated for confounding and effect modification. Results are reported as odds ratios (95% confidence interval) with *p*-values. A receiver operating characteristic curve was generated to evaluate hyperlactataemia as a predictor of TRD status.

Univariate models were evaluated for effect modification by binary ThDP level, where where a deficiency was defined as ThDP <40 nmol/L. To ensure analyses included baseline biochemical thiamine status before supplementation, thiamine biomarker analyses were excluded in children who were known to have received thiamine supplementation before the initial blood sample was taken or who had a free thiamine concentration of >90th percentile of the study sample which were assumed to have been from children who received therapeutic thiamine before the baseline biomarker blood sample was taken. Analyses with ETKac resulted in models which failed to converge. Therefore, the relationship between ETKac and hyperlactataemia was further evaluated by identifying median ETKac by binary baseline lactate status and Spearman correlation.

Analyses completed after the initial statistical analysis plan was finalised included the above ETKac analyses and use of logistic regression to examine the relationship between lactate and mortality. Additionally, a receiver operating characteristic curve was generated to evaluate hyperlactataemia as a predictor of severe thiamine deficiency by ThDP level (<40 nmol/L). Missing data were not imputed for any analysis. Analyses were performed using BlueSky Statistics (Bluesky Statistics LLC, Chicago, IL, USA) version 7.4. Statistical significance was set at an alpha level of 0.05.

## Results

**Figure 1.**
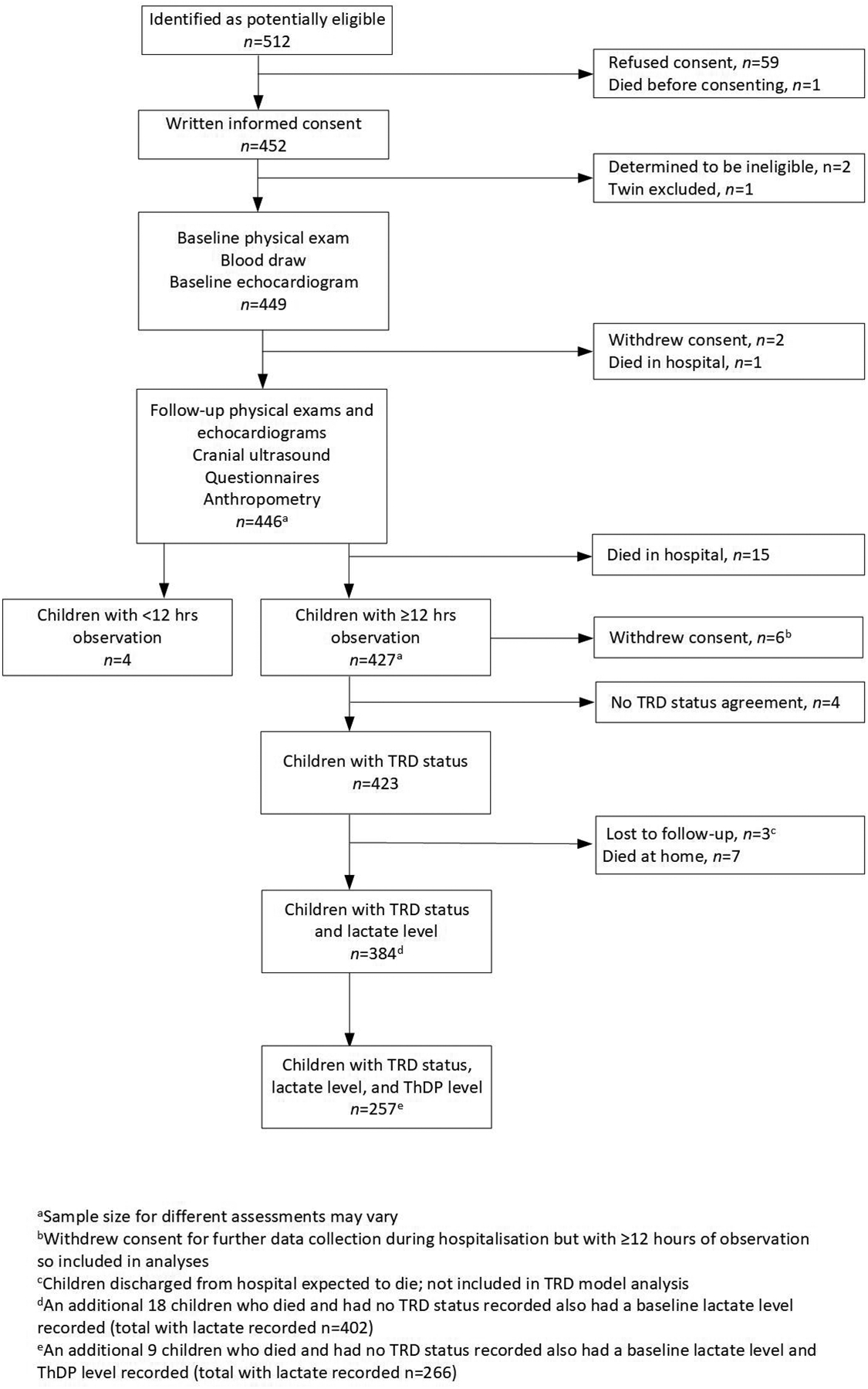
Flowchart of participant inclusion and exclusion Adapted from Smith TJ, Arnold CD, Fischer PR, et al. A predictive model for thiamine responsive disorders among infants and young children: results from a prospective cohort study in Lao People’s Democratic Republic. J Pediatr. 2024;16:113961.

The Lao Thiamine study enrolled 449 hospitalised children with at least one sign or symptom of a TDD (Figure 1). The majority of children were male (60.7%) and Hmong (61.7%) or Khmu (23.9%) by maternal ethnic group (Table 1). The median (IQR) age was 2.9 (4.0) months. Of all the children enrolled, 423 (94.2%) were assigned a TRD status: 257 (60.8%) had a positive clinical response to thiamine, and so were deemed to be ill with a TRD. Of those assigned a TRD status, 384 (90.8%) had a measured baseline blood lactate level. There were similar rates of missing lactate data in each TRD group [23 (8.9%) missing in the TRD group and 16 (9.6%) in the non-TRD group, *p*=0.8]. Additionally, in seven of 25 (28%) deceased patients, a baseline blood lactate level had not been recorded.

**Figure 2.**
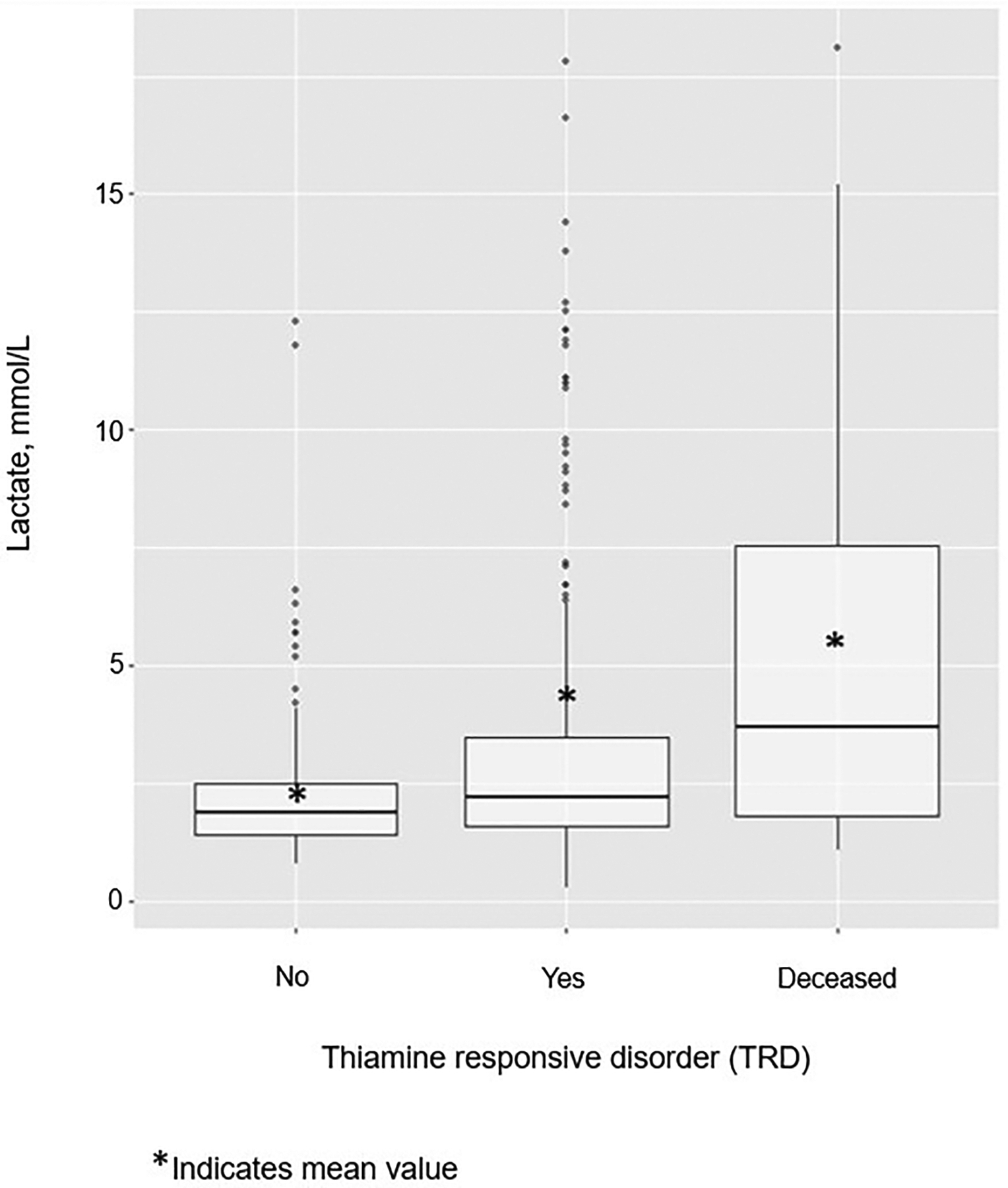
Median, mean and distribution of baseline blood lactate by thiamine responsive disorders (TRD) status. Mean (SD) baseline venous blood includes children ill without a TRD [2.36 (1.61) mmol/L], ill with a TRD [3.35 (3.06) mmol/L] or deceased and unevaluable for thiamine response [5.16 (4.91) mmol/L].

The median (IQR) baseline blood lactate level was significantly higher in children ill with a TRD *vs* non-TRD [3.35 (3.06) *vs* 2.36 (1.61) mmol/L, *p*<0.001] (Figure 2). Children with hyperlactataemia at presentation had twice the odds of a positive clinical response to therapeutic thiamine than children with reference range lactate levels [OR (95%CI) 2.32 (1.28–4.45), *p*=0.007] (Table 2). For every 1 mmol/L increase in baseline lactate, there was a 19% increase in the odds of illness with a TRD [OR (95% CI) 1.19 (1.08–1.34), *p*=0.001] (Table 2).

**Figure 3.**
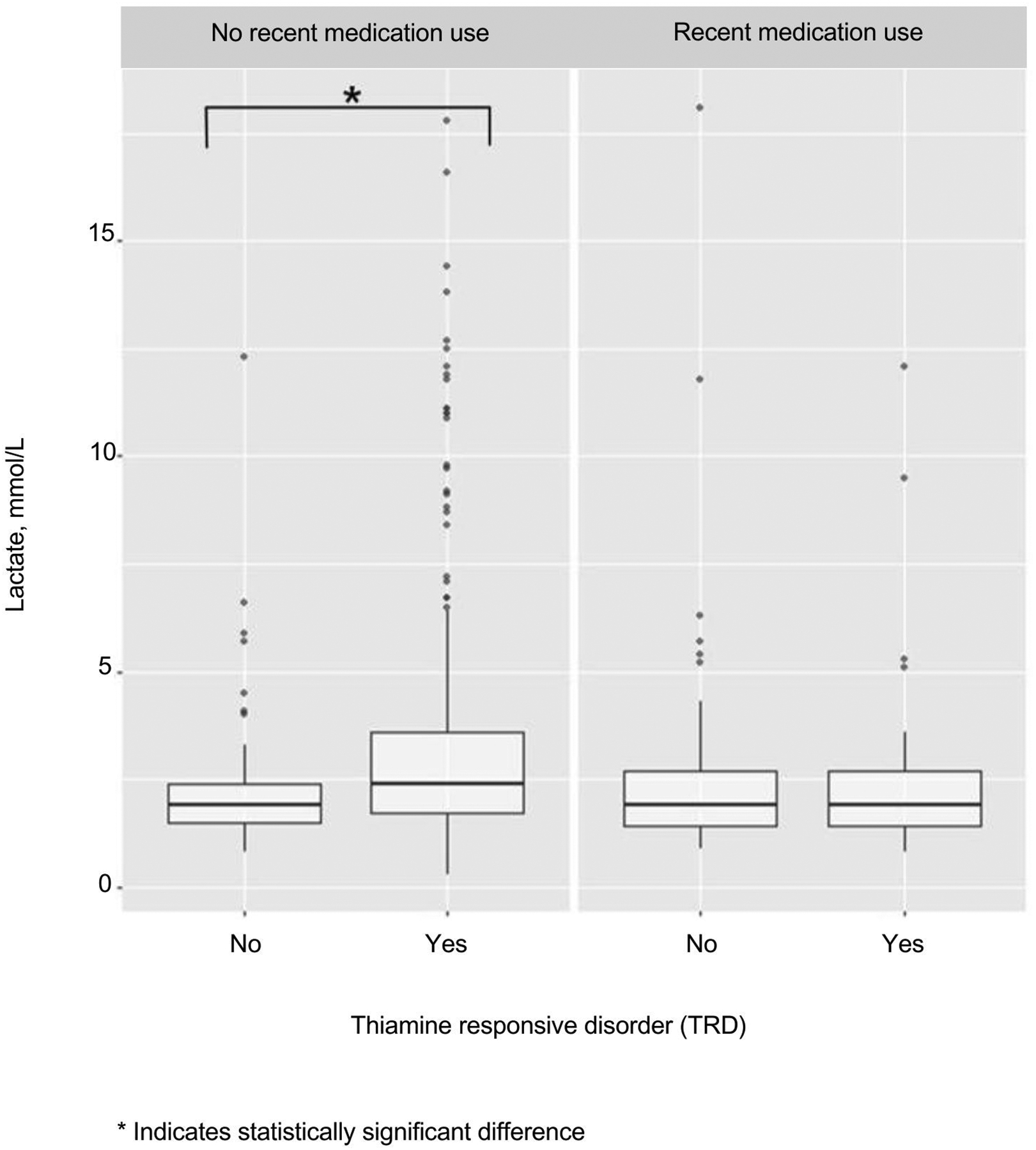
Baseline blood lactate as a predictor of thiamine responsive disorders (TRD) according to recent use of medication.

While hyperlactataemia was positively associated with TRDs overall, this relationship varied according to carer-reported administration of medication for treatment of an acute illness in the 2 weeks before hospitalisation (Figure 3). In the children who received any medication in the 2 weeks before hospitalisation, there was no difference in the odds of a favourable response to thiamine according to baseline lactate level [OR (95%CI) 0.6 (0.1–1.9), *p*=0.4] (Table 2). In children who did not receive medication during the 2 weeks before hospitalisation, hyperlactataemia was associated with increased odds of favourable response to thiamine [OR (95% CI) 4.1 (1.9–10.2), *p*=0.001] (Table 2). No other adjustment variables were found to confound or modify the relationship between lactate and TRD status.

The median (IQR) ThDP level in children with TRD *vs* non-TRD illnesses was not significantly different [71.7 (59.6) nmol/L v*s* 64.1 (49.7) nmol/L, *p*=0.07] [11]. The association between hyperlactataemia and TRD was not modified by thiamine status, with ThDP <40 nmol being defined as thiamine deficiency [OR (95% CI) 2.6 (0.8–9.2), *p*=0.13] (Table 2). However, thiamine deficiency and hyperlactataemia were associated independently of TRD status: The odds of ThDP <40 nmol/L were 10 times greater in the children with hyperlactataemia than in those with a reference range lactate level [10.6 (4.8–24.5), *p*<0.001] (Table 2).

In 232 children, both a baseline blood lactate level and ETKac were recorded, and 30 of them had baseline hyperlactataemia. The median (IQR) ETKac was greater in children with baseline hyperlactataemia *vs* reference range lactate [1.72 (0.45) *vs* 1.23 (0.31), *p*<0.001]. The Spearman correlation co-efficient demonstrated a weakly positive association between hyperlactatemia and elevated ETKac (ρ=0.22, *p*<0.001).

**Figure 4.**
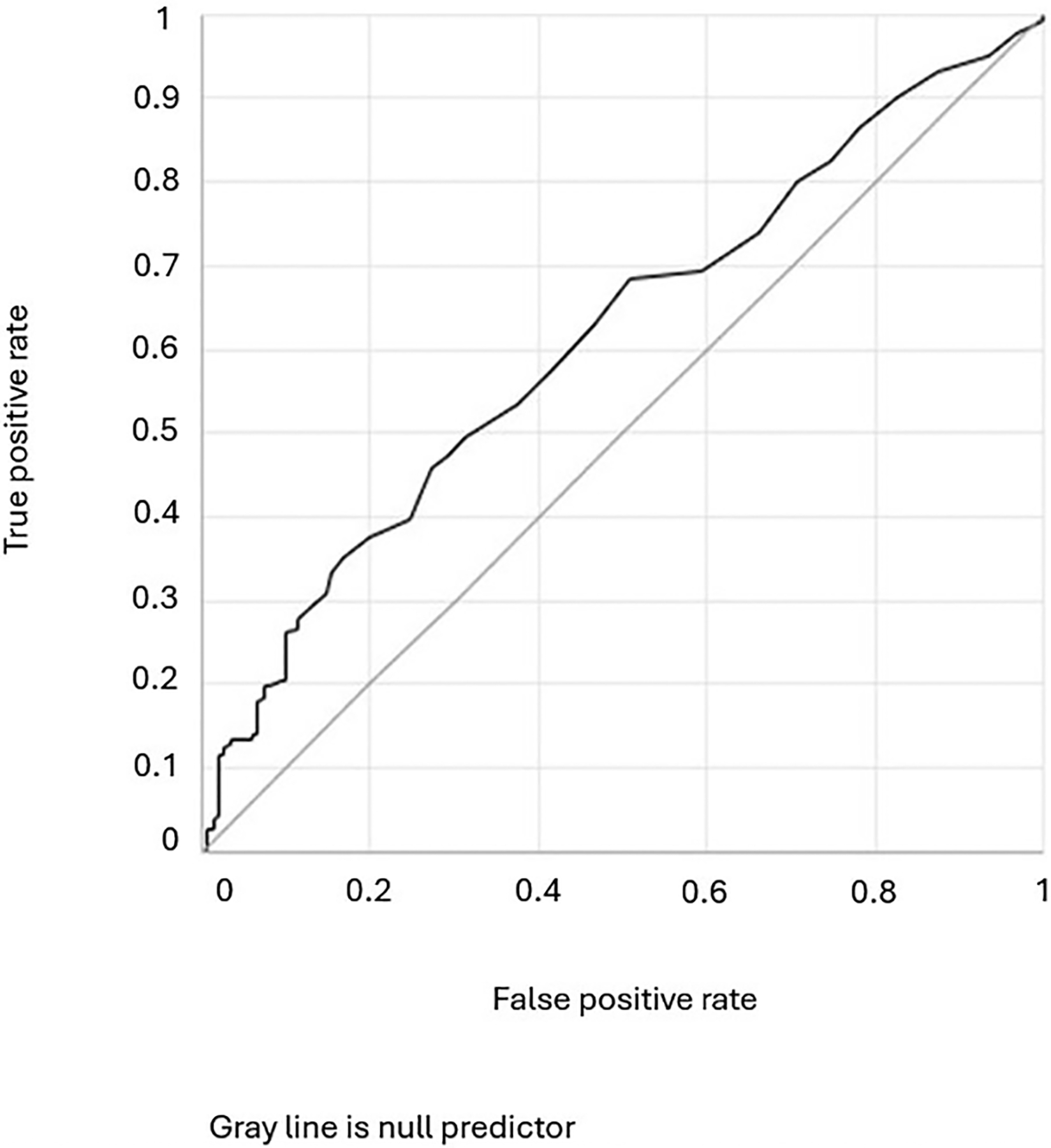
Receiver operating characteristic curve depicting performance of baseline blood lactate as a predictor of thiamine responsive disorders (TRD). AUROC (95% CI) 0.61 (0.56–0.67), *p*=0.001.

**Figure 5.**
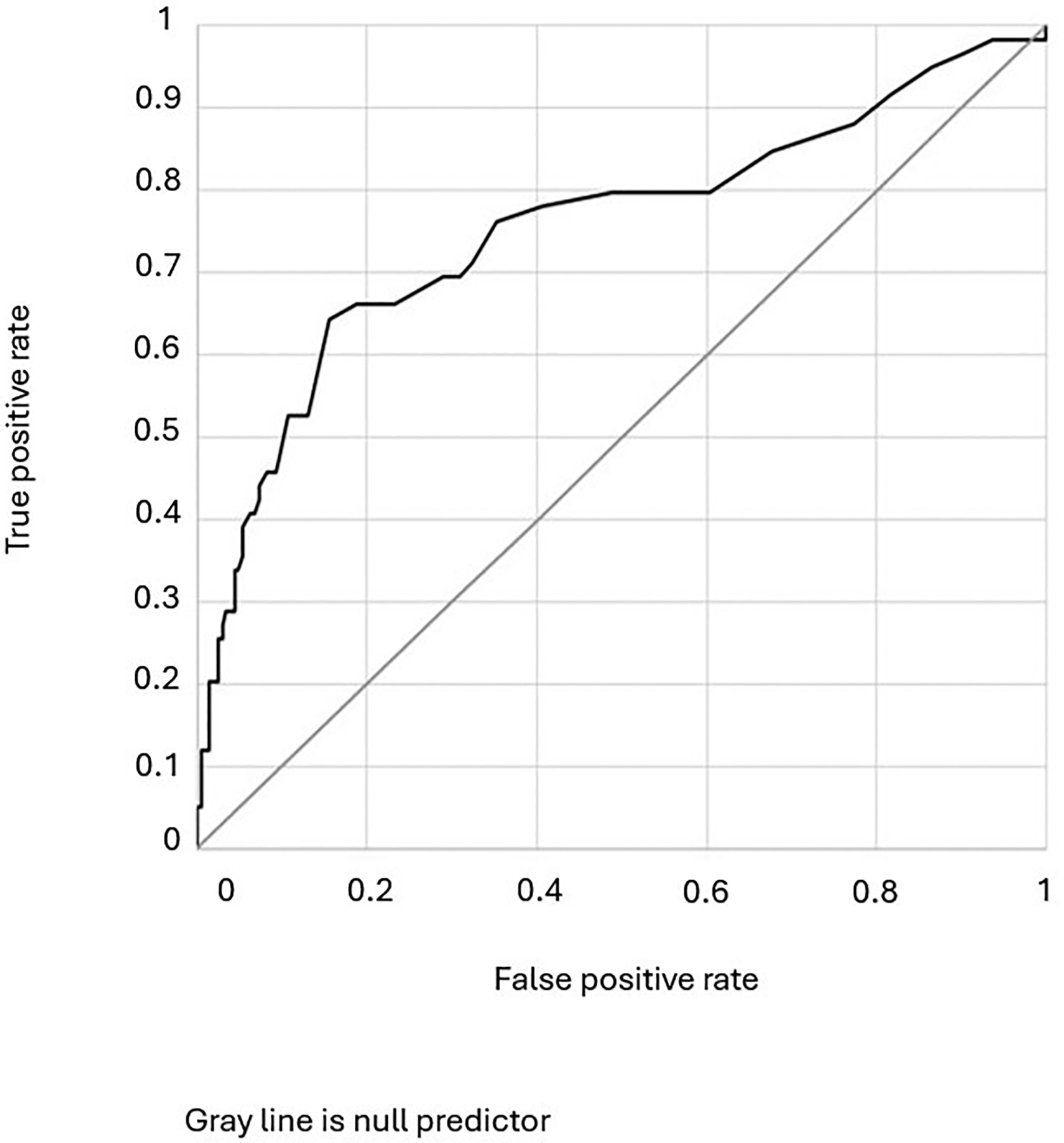
Receiver operating characteristic curve depicting performance of baseline blood lactate as a predictor of biochemical thiamine status. AUROC (95% CI) 0.76 (0.67–0.84), *p*<0.001.

Variables were also evaluated for predictive performance in receiver operating characteristic curves. Overall, baseline lactate level was a statistically significant predictor, but had relatively little additional usefulness in predicting a favourable response to therapeutic thiamine [AUROC 0.61 (0.56–0.67), *p*=0.001] (Figure 4). Notably, including carer-reported recent administration of medication in the model did not improve predictive performance. However, when baseline lactate level was modelled as a predictor of biochemical thiamine deficiency, the performance of the model improved significantly [AUROC (95% CI) 0.76 (0.67–0.84), *p*<0.001] (Figure 5).

Finally, baseline hyperlactataemia was positively associated with mortality (Figure 2). Of the children who died (total = 26, 20 died with measured baseline lactate), the median (IQR) baseline blood lactate level was 3.5 (5.9) mmol/L, with a maximum baseline level of 18.1 mmol/L. Moreover, children presenting for hospital care with baseline hyperlactataemia had almost four times greater odds of death than those with reference-range baseline blood lactate levels [OR (95% CI) 3.51 (1.38–8.94), *p*=0.009] (Table 2).

## Discussion

This study of children with signs and symptoms suggestive of TDDs in a high-risk area demonstrates that hyperlactataemia is associated with a favourable clinical response to therapeutic thiamine, biochemical thiamine deficiency, and increased odds of mortality. Considering the challenges of diagnosing TDDs on the basis of other clinical and laboratory findings, lactate may provide a clinically useful biomarker to identify children who could benefit from therapeutic thiamine [17,19–23].

The association between hyperlactataemia and a positive clinical response to thiamine demonstrated here has also been reported in children and adults with a variety of presentations of TDDs. For example, a recent study of adults in northern India with high-output heart failure demonstrated that those with a clinical presentation responsive to thiamine had a significantly higher mean lactate than thiamine non-responders [24]. In another study of adults in the same region who presented with weakness consistent with thiamine deficiency-related neuropathy, mean lactate was higher in those who responded favourably to receiving thiamine [25]. Finally, in infants in northern India with a baseline lactate level of >15 mmol/L, administration of therapeutic thiamine was followed by improvement in tachycardia, perfusion and irritability within 4 hours [26].

In this study, despite the significant and positive association between hyperlactataemia and TRDs, hyperlactataemia was present in sick children with and without a TRD. The range of normal-to-elevated lactate identified in both illness groups could be secondary to challenges in TRD group assignment. For example, a clinical response to thiamine could not be isolated from response to other treatment given to a child during hospitalisation, such as antibiotics, supplemental oxygen or fluid resuscitation [11]. Positive clinical changes secondary to concurrent treatments might have complicated the expert paediatricians’ assignment to the TRD or non-TRD group. This lack of variable separation might also explain why low ThDP did not modify the relationship between hyperlactataemia and TRDs, but did demonstrate an independent positive association with hyperlactataemia. However, further analysis of the Lao Thiamine study data also demonstrated a lack of association between clinical TRD presentation in sick children and ThDP level in matched community controls [27].

Alternatively, there might be hyperlactataemia in children with TRD and non-TRD illnesses secondary to an underlying pathophysiology which is not driven by thiamine deficiency. Other aetiologies of hyperlactataemia have been well established in the literature, including tissue hypoxia secondary to a variety of shock states, regional ischaemia, medication and seizure [6,28,29]. These secondary causes of hyperlactataemia may have occurred independently, or co-occurred in patients with thiamine deficiency. For example, in cases of Shoshin beriberi with fulminant cardiovascular collapse and shock, hyperlactataemia may be attributable to tissue hypoxia as opposed to disruption of aerobic metabolism by thiamine deficiency [30,31]. Additionally, other studies have suggested that lactate may not be a byproduct of anaerobic metabolism in tissue hypoxia primarily, but rather a purposeful supplemental energy source in the context of a stress response [6,29,32]. Therefore, hyperlactataemia may be related to a severe illness state resulting in an adrenergic stress response. This range of causes of hyperlactatemia which are potentially separate from or in addition to thiamine deficiency is supported by the finding of a significant difference in median lactate between the TRD and non-TRD illness groups, without modification by ThDP concentration, and the positive and significant but weak relationship between ETKac and hyperlactatemia in this study.

In interaction analyses, the overall positive association between hyperlactataemia and TRDs was found to vary on the basis of caregiver-reported administration of medication for an acute illness in the 2 weeks before hospitalisation. Specifically, hyperlactataemia only predicted TRD status in children who had not recently received medication for an acute illness. The underlying pathology of acute *vs* subacute TDDs might be an explanation. For example, fulminant cardiovascular collapse and hyperlactataemia are reported in Shoshin beriberi, whereas hyperlactataemia is not consistently described in clinical manifestations of subacute ‘wet’ or cardiac beriberi [8,9]. Thus, despite commonality in the cardiac manifestations of both wet and Shoshin beriberi, the acute *vs* subacute timeline of cardiac defects might indicate a difference in the pathological process reflected in differences in hyperlactataemia.

Finally, the positive association between hyperlactataemia and mortality is consistent with other aetiologies of severe illness [2]. However, this relationship has been shown to be disease-dependent in that lactate elevation predicts mortality more consistently in some disease processes than in others [4–6]. For example, hyperlactataemia was strongly associated with mortality in post-cardiac arrest patients but had little relevance for prognostication in patients with uncomplicated diabetic ketoacidosis [6]. Therefore, this study contributes to the finding that hyperlactataemia is associated with mortality in infants and children hospitalised for the management of symptoms indicating TDDs. Trending lactate levels might be useful prognostically as trending has been used to monitor response to treatment in other critical illnesses [1]; decreasing lactate has been associated with clinical improvement in adults with thiamine deficiency and heart failure [24].

This is the first study to examine the relationship between lactate and clinical responsiveness to the administration of thiamine in young children presenting for hospital care for signs and symptoms suggesting TDDs. Limitations include the use of venous blood lactate as opposed to the gold standard of arterial sampling, lack of lactate measurement after thiamine administration and subjectivity of defining clinical response to thiamine by an expert panel of paediatricians [11]. Additionally, for analyses including ThDP, patients who received thiamine before baseline blood sampling and/or who had a free thiamine concentration of >90th percentile were excluded from analysis, reducing the sample size. This exclusion included children who received thiamine at a health centre before referral to hospital and/or more severe, probably life-threatening cases who required immediate administration of thiamine on admission to hospital. Therefore, the missing data represent the more severe clinical presentations and limit the dataset in these analyses to sick but more mildly affected children.

In summary, hyperlactataemia and a favourable clinical response to the therapeutic administration of thiamine are positively associated. Although thiamine deficiency defined by the level of ThDP did not modify the relationship between hyperlactataemia and TRDs, ThDP and hyperlactataemia were independently associated, suggesting the usefulness of lactate in identifying children who might benefit from receiving thiamine. Finally, the association between hyperlactataemia and mortality suggests that children in areas at high risk of severe thiamine deficiency who present with hyperlactataemia might require special medical attention throughout their clinical course because of the increased odds of mortality. To improve clinical outcome in children with hyperlactataemia and thiamine-responsive disorders, future studies should focus on the relationship between specific features of physical examination or imaging studies, such as echocardiogram or brain ultrasound, and baseline hyperlactataemia and the change in lactate level after administration of therapeutic thiamine.

## Supplementary Material

1

## Figures and Tables

**Table 1.** Baseline characteristics.

Characteristic	Enrolled children (*n*=449)	Subset lactate recorded (*n*=402)	Subset lactate and ThDP recorded (*n*=266)	*p*-value
Age, mths	2.90 (4.00)	2.90 (3.95)	4.86 (3.69)	<0.001
Male	272 (60.7)	244 (60.7)	162 (60.9)	0.92
Residential province				
Luang Prabang	411 (91.5)	367 (91.3)	240 (90.2)	0.24
Oudomxay	21 (4.7)	19 (4.72)	15 (5.6)	0.25
Missing	17 (3.8)	16 (3.98)	11 (4.1)	0.65
Maternal ethnic group				
Hmong	274 (61.0)	242 (60.2)	159 (59.8)	0.38
Khmu	107 (23.8)	93 (23.1)	58 (21.8)	0.19
Lao	49 (10.9)	49 (12.2)	36 (13.5)	0.036
Other	11 (2.5)	11 (2.74)	10 (3.75)	0.032
Missing	8 (1.8)	7 (1.74)	3 (1.13)	<0.001
Breastfeeding status	*n*=323	*n*=293	*n*=179	
Exclusively breastfed (age <6 months)	252 (78.0)	225 (76.8)	129 (72.1)	0.13

Age is reported as median (IQR). All other characteristics are reported as number (%) of participants per category. Enrolled Children includes all who presented for hospital care with at least one sign/symptom of a TDD and were enrolled in the study. Subset Lactate Recorded includes only children for whom a baseline lactate value was recorded. Subset Lactate and ThDP Recorded includes only children for whom a baseline lactate value and baseline ThDP were recorded. The difference in participant characteristics between Enrolled Children and Subset Lactate Recorded did not reach statistical significance for any characteristic. The difference in characteristics between Enrolled Children and Subset Lactate and ThDP Recorded as determined by Student’s *t*-test or the χ^2^ test is reported as a *p*-value.

**Table 2.** Univariable models predicting outcome by baseline blood lactate concentration and multivariable models predicting thiamine responsive disorders (TRD) with interaction terms.

Predictor	Interaction subset	OR (95% CI)	*p*-value
Binary lactate	Interaction term	0.14 (0.026–0.60)	0.0083
	No recent medication	4.07 (1.87–10.20)	0.0010
	Recent medication	0.55 (0.14–1.94)	0.37
Continuous lactate	Interaction term	0.70 (0.53–0.88)	0.0038
	No recent medication	1.37 (1.18–1.67)	0.001
	Recent medication	0.96 (0.78–1.13)	0.61
Binary lactate	Interaction term	2.55 (0.46–14.66)	0.29
	ThDP ≥40 nmol/L	1.00 (0.29–3.58)	1.00
	ThDP <40 nmol/L	2.55 (0.80–9.18)	0.13
Continuous lactate	Interaction term	1.41 (0.90–2.23)	0.13
	ThDP ≥40 nmol/L	1.19 (0.92–1.60)	0.22
	ThDP <40 nmol/L	1.31 (1.03–1.83)	0.060
Predictor	Outcome	OR (95% CI)	*p*-value
Binary lactate	Ill with a TRD	2.32 (1.28–4.45)	0.007
Binary lactate	ThDP <40 nmol/L	10.6 (4.8–24.5)	<0.001
Binary lactate	Death	3.51 (1.38–8.94)	0.009

## Data Availability

The dataset analysed in this secondary analysis can be found at: https://doi.org/10.17605/OSF.IO/JFKE3.

## Abbreviations:

AUROC: area under the receiver operating curve
ETKac: erythrocyte transketolase activity coefficient
IRB: institutional review board
IQR: interquartile range
Lao PDR: Lao People’s Democratic Republic
TDD: thiamine deficiency disorder
ThDP: thiamine diphosphate
TRD: thiamine responsive disorder
