# Supplementary material for: Relationship between lactate and thiamine-responsive disorders in hospitalised infants and children in Lao PDR: secondary analysis of a prospective cohort study
Source: Paediatr Int Child Health. Author manuscript; Available in PMC 2024 Nov 22. (PMC11581904; doi:10.1080/20469047.2024.2421624)
Supplement: 1 [file NIHMS2031618-supplement-1.pdf]

Supplemental Table 1. Definition of variables: thiamine responsive disorders (TRD), baseline vital signs, physical exam features, biochemical markers, and regression model adjustment variables.

Variable	Definition
TRD status	Positive clinical response to thiamine: illnesses classified as classical beriberi or probable TRD.<br>Thiamine non-responders: illnesses classified as possible TRD or not likely TRD.
Baseline vital signs and physical examination features	
Tachypnoea	Respiratory rate<br>>60/min for ages 3–8 weeks<br>>50/min for ages 2–11 months<br>>40/min for ages 12–18 months
Tachycardia	Heart rate<br>>160/min for ages <12 months<br>>120/min for ages 12–18 months
Low pulse oximeter reading	Pulse oximeter reading <92%
Low oxygenation status	Oxygen saturation <92%, or receiving supplemental oxygen regardless of pulse oximeter reading
Oedema	Presence of any peri-orbital oedema, peripheral oedema or anasarca
Liver enlargement	Liver palpated >2 cm belowthe right costal margin on supine examination
Difficulty breathing	Any chest indrawing, nasal flaring or noisy breathing
Refusal to feed	Refusal to breastfeed or refusal of infant formula or food for more than 24 hours
Vomiting	Repetitive or recurrent vomiting with no obvious other cause (i.e. vomiting >3 times in the past 24 hours)
Persistent crying	Crying not relieved by soothing or feeding with no obvious other cause

Voice change	Hoarse voice or cry or loss of voice
Abnormal eye movements	Nystagmus or other unusual eye movements
Muscle twitching	Unintentional muscle movements
Convulsion	Sudden, irregular and involuntary movement of a limb or the body
Opisthotonus	Spasm of the muscles causing backward arching of the head, neck and spine or other abnormal posturing
Unresponsiveness	Lack of alertness or response to stimulation by voice or pain
Biochemical variables measured at baseline evaluation	
Blood lactate level	Reference range ≤4.0 mmol/L<br>Elevated >4.0 mmol/L
Blood thiamine diphosphate (ThDP) level	Thiamine sufficient: ThDP ≥40 nmol/L<br>Thiamine deficient: ThDP <40 nmol/L
Blood erythrocyte kinase activity coefficient (ETKac) level	Thiamine sufficient: ETKac >1.25 U/g haemoglobin<br>Thiamine deficient: ETKac ≤1. 25 U/g haemoglobin
Adjustment variables	
Age	Age in months
Sex	Male or female sex assigned at birth
Province	Self-reported home province
Maternal ethnic group	Self-reported ethnic group by mother, including Hmong, Khmu, Lao, Lue or other
Vomiting	Caregiver-reported presence or absence in the 2 weeks before hospitalisation. Evaluated for presence in mother or child, separately.

Diarrhoea	Caregiver-reported presence or absence in the 2 weeks before hospitalisation. Evaluated for presence in mother or child, separately.
Recent illness	Caregiver-reported presence or absence in the 2 weeks before hospitalisation. Evaluated for presence in mother or child, separately.
Recent medication	Caregiver-reported consumption of any medication such as Tylenol for management of an illness in the 2 weeks before hospitalisation. Evaluated in mother or child, separately.
